# Generating Natural, Intelligible Speech From Brain Activity in Motor, Premotor, and Inferior Frontal Cortices

**DOI:** 10.3389/fnins.2019.01267

**Published:** 2019-11-22

**Authors:** Christian Herff, Lorenz Diener, Miguel Angrick, Emily Mugler, Matthew C. Tate, Matthew A. Goldrick, Dean J. Krusienski, Marc W. Slutzky, Tanja Schultz

**Affiliations:** ^1^School of Mental Health & Neuroscience, Maastricht University, Maastricht, Netherlands; ^2^Cognitive Systems Lab, University of Bremen, Bremen, Germany; ^3^Department of Neurology, Northwestern University, Chicago, IL, United States; ^4^Department of Neurosurgery, Northwestern University, Chicago, IL, United States; ^5^Department of Linguistics, Northwestern University, Chicago, IL, United States; ^6^Biomedical Engineering Department, Virginia Commonwealth University, Richmond, VA, United States; ^7^Department of Physiology, Northwestern University, Chicago, IL, United States; ^8^Department of Physical Medicine & Rehabilitation, Northwestern University, Chicago, IL, United States

**Keywords:** ECoG, BCI, brain-computer interface, speech, synthesis, brain-to-speech

## Abstract

Neural interfaces that directly produce intelligible speech from brain activity would allow people with severe impairment from neurological disorders to communicate more naturally. Here, we record neural population activity in motor, premotor and inferior frontal cortices during speech production using electrocorticography (ECoG) and show that ECoG signals alone can be used to generate intelligible speech output that can preserve conversational cues. To produce speech directly from neural data, we adapted a method from the field of speech synthesis called unit selection, in which units of speech are concatenated to form audible output. In our approach, which we call *Brain-To-Speech*, we chose subsequent units of speech based on the measured ECoG activity to generate audio waveforms directly from the neural recordings. *Brain-To-Speech* employed the user's own voice to generate speech that sounded very natural and included features such as prosody and accentuation. By investigating the brain areas involved in speech production separately, we found that speech motor cortex provided more information for the reconstruction process than the other cortical areas.

## Introduction

Brain-computer interfaces (BCIs; Wolpaw et al., 2002) that process natural speech present a very intuitive paradigm for direct machine-mediated human communication and have the potential to restore intuitive communication for people unable to speak due to paralysis. In recent years, impressive advances in the decoding of speech processes from neural signals have been achieved. Electrocorticographic (ECoG) signals recorded from the cortical surface are well-suited for this purpose due to the broad coverage of multiple cortical areas (Herff and Schultz, 2016). Using ECoG, laryngeal activity (Dichter et al., 2018), phonetic features (Mesgarani et al., 2014; Lotte et al., 2015), articulatory gestures (Chartier et al., 2018; Mugler et al., 2018), phonemes (Mugler et al., 2014; Ramsey et al., 2017), words (Kellis et al., 2010; Milsap et al., 2019), and continuous sentences (Herff et al., 2015; Moses et al., 2016, 2018) have been investigated. To provide speech-impaired patients with the full expressive power of speech, it is crucial to include acoustic, prosodic, and linguistic cues. These cues include, but are not limited to, pitch (intonation), timing, stress, emphasis, and pauses, which are required to discriminate statements from questions, differentiate words and meaning, carry emotions, and to convey humor and sarcasm, to name only a few. Furthermore, the decoding of sentences or words into textual representations always introduces a delay of at least the length of the smallest recognizable speech unit, which could potentially lead to severe articulatory disturbances (Stuart et al., 2002) when playing back the delayed audible feedback to the user. In contrast, the direct conversion of brain activity into audible speech can enable natural conversation, as it can provide rapid auditory feedback.

The speech production process has been widely studied (Tian and Poeppel, 2010; Tourville and Guenther, 2011; Hickok, 2012), and while it is not fully understood, a number of brain areas are known to be involved at the level of producing articulation. These areas include the inferior frontal gyrus (Okada et al., 2018), the pre-motor cortex (Glanz et al., 2018), and the speech motor cortex (Bouchard et al., 2013; Ramsey et al., 2017). Other areas such as superior temporal gyrus also show activity during speech production (Kubanek et al., 2013; Brumberg et al., 2016), but it is unclear whether these areas are involved in articulatory or semantic processing.

Previous studies have reconstructed perceived audio from ECoG (Pasley et al., 2012) and spectrotemporal modulations of real-life sounds from fMRI (Santoro et al., 2017). Martin et al. reconstructed spectrotemporal features of speech from speech production and perception areas (Martin et al., 2014), but did not synthesize audio waveforms from these features. Akbari and colleagues extended these findings and synthesized high quality audio from cortical areas involved in speech perception using Deep Neural Networks (Akbari et al., 2019). In an online study in motor-intact patients, Leuthardt and colleagues demonstrated one-dimensional cursor control using ECoG activity during the production of two isolated phonemes (Leuthardt et al., 2011). The first study presenting real-time, closed-loop synthesis of speech from cortical spikes in a paralyzed patient demonstrated accurate reconstruction of formant frequencies in attempted vowel production (Guenther et al., 2009) and thereby laid the foundations for speech neuroprostheses.

Recently, two different approaches synthesizing speech from neural activity during speech production have been presented. Both achieve very high quality audio by employing deep neural networks and an intermediate representation of speech, one study uses articulatory representations of the speech production process (Anumanchipalli et al., 2019), which are then transformed into audio output, the other (performed on the same dataset as this study) transforms the neural recordings to a spectral representation first, which is then transformed to an audio waveform with a second neural network (Angrick et al., 2019).

Here, we present an alternative approach which directly reconstructs intelligible, naturalistic speech (that is, speech with prosody and accentuation) from speech-related motor cortical activity using a very simple pattern matching approach from the speech synthesis community. The presented approach is simple to implement, requires little training data, is real-time ready, and does not require the design of deep learning architectures.

## Materials and Methods

###  Experiment Design

**Figure 1:**
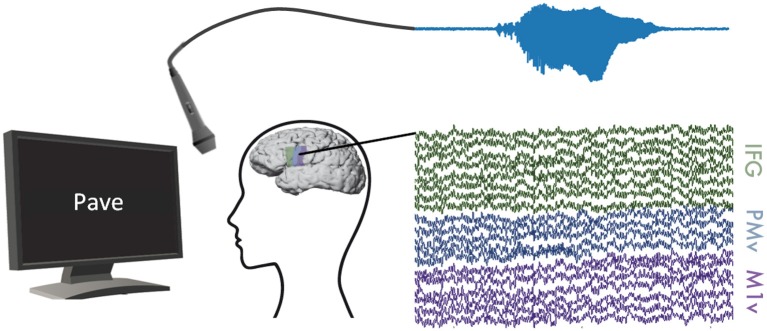
Experimental Setup: ECoG and audible speech (light blue) were measured simultaneously while participants read words shown on a computer screen. We recorded ECoG data on inferior frontal (green), premotor (blue), and motor (purple) cortices.

Participants in our study were asked to read words shown to them on a computer screen aloud (Figure 1). Most presented words were monosyllabic and followed a consonant-vowel-consonant (CVC) structure. This set of words primarily comprised of the Modified Rhyme Test presented in House et al. (1963) and supplemented with additional words to better reflect the phoneme distribution of American English (Mines et al., 1978). Words were displayed one at a time at rate of one word every 2 s in a randomized order. Participants read between 244 and 372 words resulting in 8.3 to 11.7 min of recordings each. Table 1 summarizes recording length (in seconds) and number of repeated words for all participants. The data used in this study were also used in Mugler et al. (2018) and Angrick et al. (2019).

**Table 1:** Participant demographics and electrode information.

**Participant**	**#Words**	**Recording time (s)**	**#IFG**	**#PMv**	**#M1v**
1	368	752.8	12	19	18
2	370	761.7	8	15	19
3	249	509.2	16	21	20
4	249	571.5	11	29	18
5	244	499.2	0	19	19
6	372	760.8	15	18	12

###  Participants

Patients undergoing awake craniotomy with cortical stimulation and recording as part of normal clinical care were selected for enrollment. All participants gave written informed consent to participate in the study prior to surgery. The study design was approved by the Institutional Review Board of Northwestern University. We recorded ECoG activity from six patients (1 female, 55.5 ± 10.1 yo) undergoing awake craniotomies for glioma resection. Tumors locations lay at least two gyri (2–3 cm) away from the recording sites. All participants were native English speakers.

###  Cortical Mapping

All participants were implanted with grids on the left hemisphere. The experimental grids were specifically placed to cover areas involved in the speech production process. Electrode grids were placed based on functional responses to cortical stimulation and on anatomical mapping. Final locations were confirmed using intraoperative navigation software (Brainlab), preoperative MRI, and intraoperative photography (Hermes et al., 2010).

To map the eloquent cortex, electrocortical stimulation was used. Areas producing speech or anomia arrest during stimulation were labeled as language associated, while areas producing movement of tongue and articulators during stimulation were labeled as functional speech motor areas.

**Figure 2:**
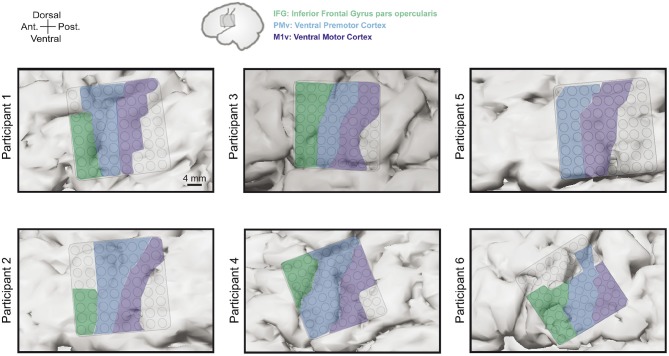
Electrode grid positions for all six participants. Grids always covered areas in inferior frontal gyrus pars opercularis (IFG, green), ventral premotor cortex (PMv, blue), and ventral motor cortex (M1v, purple).

Grid locations were different for each participants based on craniotomy location but always covered targeted areas of ventral motor cortex (M1v), premotor cortex (PMv), and inferior frontal gyrus pars opercularis (IFG). Since there is no clear cytoarchitectural difference between M1v and PMv, we defined PMv as the anterior half of the precentral gyrus and M1v as the posterior half of the precentral gyrus. Table 1 provides information about the number of electrodes in each specific region for each participant. Grid locations for our six participants can be found in Figure 2.

###  Data Recording

We recorded ECoG using an 8 x 8, 64-channel electrode grid (Integra, 4 mm spacing) and a Neuroport data acquisition system (Blackrock Microsystems, Inc.). ECoG data was sampled at 2 kHz and bandpass-filtered between 0.5 and 300 Hz.

Audio data was recorded in parallel using a unidirectional lapel microphone (Sennheiser) and wirelessly transmitted to a recording station (Califone). Audio data was sampled at 48 kHz. Stimulus presentation and synchronous data recording was facilitated using BCI2000 (Schalk et al., 2004).

###  ECoG Signal Processing

To extract meaningful information from the recorded ECoG activity, we extracted logarithmic high-gamma power. The gamma-band is known to reflect ensemble spiking (Ray et al., 2008) and contain localized information for motor (Miller et al., 2007) and speech (Crone et al., 2001; Leuthardt et al., 2012) tasks. To remove slow drifts in the data, we first applied linear detrending to the raw ECoG data. The signal was then downsampled from 2 kHz to 600 Hz to reduce dataset size. We then forward-backward filtered the signals of all 64 electrodes using elliptic IIR low-pass (170 Hz cut-off, filter order 14) and high-pass (70 Hz cut-off, filter order 13) filters to represent the high-gamma band. To reduce the first harmonic of the 60 Hz line noise, we applied an elliptic IIR notch filter (118–122 Hz, filter order 13). Logarithmic high-gamma power was calculated by taking the logarithm of the squared signal. As the speech production process includes complex temporal dynamics (Sahin et al., 2009; Brumberg et al., 2016), a 450 ms long window centered on the current sample was considered and downsampled to 20 Hz. The resulting matrix of 64 channels × 9 time points was flattened to form a feature vector of 64 channels × 9 time points = 576features. Extracted features were normalized to zero mean and unit variance. To capture the fast dynamics of speech, a new feature vector was extracted every 10 ms. We generated speech using either all 64 electrodes or the electrodes from individual areas separately (IFG, PMv and M1v, mean of 12.4, 20.2 and 17.7 electrodes, respectively).

###  Audio Signal Processing

We downsampled the recorded audio data to 16 kHz and extracted raw waveforms in 150 ms windows centered on the corresponding frame of ECoG data. Windows were extracted with a 10 ms frameshift to maintain alignment to the intervals of neural activity. We extracted the 150 ms long windows using Hanning window functions to guarantee smooth transitions (Wu et al., 2013) even with the large overlap between neighboring windows. Each of these 150 ms windows of raw audio data were considered as one speech unit in our decoding approach. Due to the long speech unit size in combination with the windowing function, no problems with pitch synchronization arise, so more complex approaches such as pitch-synchronous overlap-add (PSOLA, Moulines and Charpentier, 1990) provided no increase in reconstruction quality. The shorter speech unit length in the audio data, as compared to the high-gamma windows, was chosen as it provides a good compromise between smoothness of output and capability to capture the fast dynamics of speech. The direct mapping between speech units and corresponding high-gamma windows is necessary for our reconstruction approach.

###  Decoding Approach

We reconstructed natural audio from the measured ECoG activity by applying a technique from the speech synthesis community called unit selection (Hunt and Black, 1996). Unit selection was originally used in text-to-speech (TTS) synthesis of audio waveforms and relies on selecting and concatenating well-fitting units of speech from a large training database of speech units. The same approach was later used for voice conversion (Sundermann et al., 2006), where speech of one person is transformed to the voice of another speaker. Further extending upon this idea, unit selection was used in electromyography (EMG)-based silent speech interfaces (Zahner et al., 2014), where facial muscle movements are transformed into an audio waveform. The same approach can also be applied to other types of silent speech interfaces (Schultz et al., 2017). In all unit selection approaches, the next speech unit to concatenate to the output is chosen based on two different cost terms. The first one is how well the speech unit fits the current input, being the current phoneme in TTS or the current frame of EMG activity. This cost term is referred to as the *target cost*. The second cost function estimates how well the speech unit fits the previously selected speech units and is usually referred to as *concatenation cost*. Optimizing both cost functions together requires an iterative algorithm such as Viterbi decoding (Lou, 1995). Unit selection is known to perform well for small amounts of data, as is the case in our study. Limited datasets might not be sufficient to train more complex models with many free parameters.

**Figure 3:**
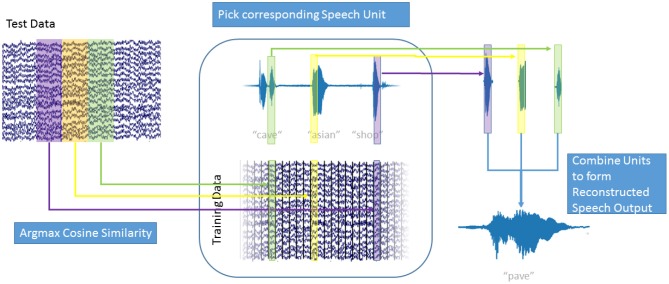
Speech Generation Approach: For each window of high gamma activity in the test data (top left), the cosine similarity to each window in the training data (center bottom) was computed. The window in the training data that maximized the cosine similarity was determined and the corresponding speech unit (center top) was selected. The resulting overlapping speech units (top right) were combined using Hanning windows to form the generated speech output (bottom right). Also see Supplementary Video 1.

In our decoding approach, we used unit selection to select the best fitting unit of speech, based on the high-gamma ECoG feature vectors (Figure 3). Our speech units were 150 ms intervals of plain audio waveforms extracted using a Hanning window function. To make sure that we selected speech units based only on the neural data and do not include any semantic information, we disregarded the *concatenation cost* for this proof-of-concept study. This speeds up the decoding process as new speech units can be selected based only on the current frame of high-gamma activity. Additionally, this allowed us to reformulate the selection approach as a maximization problem to find the ECoG feature vector $\hat{B}$ in the training data, that has the highest similarity with the current feature vector *A*:

(1) $$\hat{B}=\text{arg }\underset{B}{\text{max}}\{\text{similarity}(\text{A},\text{B})\}$$

As ECoG data and audio data are aligned, the corresponding speech unit to $\hat{B}$, from the training data, could then be selected. Figure 3 explains the decoding process: For each window of high-gamma power in the test data (top right), the cosine similarity with every window in the training data (bottom center) was computed. For the training data window *A* of high-gamma power with the highest cosine similarity to the test window $\hat{B}$, the corresponding speech unit of audio data in the training data (top center) was chosen. This process is repeated for all intervals in the test data. The chosen speech units (top right) were combined to form the generated speech (bottom right). The strongly overlapping audio data were combined by simply adding the waveforms; the Hanning windowing ensures that the resulting output is smooth. This approach is agnostic to categories of speech, such as phones, or any syntactic and semantic knowledge. It simply chooses the best fitting speech unit out of over 50,000 units (500 s / 0.01 s frameshift) instead of choosing a generalized representation, such as a phoneme or even word. This way, the speech unit with the best matching prosody and accentuation is chosen and no labeling of the data with regards to phonemes, or words is used or necessary. As our approach concatenates units of natural speech, it conserves the spectrotemporal dynamics of human speech.

This decoding approach can be likened to a very simple pattern matching approach or nearest-neighbor regression, but provided superior results than more complex approaches for our limited dataset size.

While a number of different similarity measures can be used, we applied the cosine similarity that has proven to provide good results in a number of document clustering (Steinbach et al., 2000) and computer vision applications (Nguyen and Bai, 2010). The cosine similarity between vectors *A* and *B* is defined as :

$$\text{similarity}(\text{A},\text{B})=\frac{\text{A }\cdot \text{ B}}{\left\|\text{A}\|\|\text{B}\right\|}=\frac{\sum_{i=1}^{n}A_{i}B_{i}}{\sqrt{\sum_{i=1}^{n}A_{i}^{2}}\sqrt{\sum_{i=1}^{n}B_{i}^{2}}}$$

The cosine similarity is invariant to gamma scaling, only the power distribution between electrodes influences the similarity score. By precomputing the Euclidean norm $\|\text{B}\|=\sqrt{\sum_{i=1}^{n}B_{i}^{2}}$ for all feature vectors in the training data, the cosine similarity can be computed fast enough on standard hardware to allow for real-time decoding for our data set sizes. This can be further sped up by clustering speech units together (Black and Taylor, 1997) resulting in fewer comparisons necessary. Once the high-gamma feature vector with the highest cosine similarity $\hat{B}$ was found, the corresponding speech unit in its original waveform was concatenated to the reconstructed output.

We applied our unit selection approach in a 5-fold cross-validation manner in which in each iteration 80% of the data were used as training data and the remaining 20% as testing data until all data were used as the test set exactly once. The set of spoken words in training and test set were always disjoint. To reduce the feature space, we used principal component analysis to select principal components that explain at least 70% of the total variance in the ECoG training data. The same feature space compression was than applied to the testing data, as well. This approach selected 108.1 ± 36.3 components for all electrodes, 15.9 ± 9.7 for IFG, 44 ± 15.5 for PMv, and 41.53 ± 6.8 for M1v.

###  Randomization Tests

To establish a baseline for our decoding approach, we used a randomization approach. Instead of using the speech unit corresponding to the high-gamma feature vector with the highest cosine similarity, we picked a random speech unit in the randomization condition. We combined the speech units in the same manner as the real decoding approach. We repeated this approach 1,000 times for each participant to establish a baseline of randomized reconstruction. We denoted the maximum of these randomizations as chance level in **Figure 6B**.

###  Correlation Analysis

To compare original and reconstructed audio waveforms, we transformed the waveforms into the spectral domain. This was done in 50 ms windows with 10 ms overlap. To only judge the frequency information that is important to human listeners, we transformed the magnitude spectrograms onto the mel-scale (Stevens et al., 1937) using 40 overlapping triangular filter banks. A logarithm was then applied to bring the distribution of spectral coefficients closer to a normal distribution. Pearson correlation coefficients were then computed between the original and reconstruction for each logarithmic mel-scaled coefficient. We calculated the correlations for each word individually. Significance levels are established if resulting correlations were larger than 95, 99, or 99.9% of the randomized controls, respectively.

Averaging over all 40 logarithmic mel-scaled coefficients we can look at overall correlation coefficients for the reconstruction for each of the participants. **Figure 6A** shows correlation coefficient for all participants using all electrodes, only IFG electrodes, only PMv electrodes and only M1v electrodes.

###  Listening Tests

To evaluate the intelligibility of our synthesized audio, we conducted an online forced-choice listening test with 55 (15 female) healthy volunteers. In the test, volunteers heard the 30 synthesized words with the highest spectral correlations and were given four options, the correct answer plus three distractors, to choose from. Volunteers had to pick the option which they thought the synthesized audio resembled the most. One of the four answers always needed to be selected (forced-choice). Distractor words were chosen randomly from the complete set of words used in our study, resulting in similar word length (as most words follow the CVC structure) and similar distribution of phonemes. Word order and the order of the options was randomized for each volunteer individually. We used the beagleJS framework (Kraft and Zölzer, 2014) to build our listening test.

After the listening test, we asked the volunteers to give information about their gender (15 female, 40 male), age (34.9 ± 14.1) and whether they were English native speakers (27 native speakers).

All volunteers achieved accuracies well above chance level in identification of the correct word (66.1% ± 6%) with relative low variance. These results show that our approach is very promising to generate natural, intelligible output for future voice prosthesis from neural data.

###  Objective Intelligibility Measure

In addition to the subjective listening tests, we calculated an objective intelligibility measure, namely the short-term objective intelligibility (STOI) measure (Taal et al., 2011) that is known to correlate well with subjective intelligibility. The STOI employs simple discrete Fourier transformation-based Time-frequency-decomposition. The STOI score (ranging from 0 to 1) can be mapped to an subjective intelligibility probability *d* in a transcription intelligibility test (ρ = 0.95). Taal et al. (2011) provides the formula:

$$STOI=\frac{100}{1+exp\left(ad+b\right)}$$

with *a* = −13.1903 and *b* = 6.5192. Reformulating this, we can estimate the subjective intelligibility probability *d* in a transcription intelligibility test given the calculated *STOI* with:

$$d=\frac{\log_{e}\left(\frac{100}{STOI}-1\right)-b}{a}$$

Objective measures of intelligibility, as well as spectral correlations, are notoriously unreliable in judging speech synthesis output for its intelligibility, we therefore believe our listening test provides a more realistic estimation of intelligibility for our data set, but report the STOI values for completeness. As our approach does not operate in the cepstral domain, we do not report Mel Cepstral Distortion (MCD) measures, which suffer from the same limitations as correlations.

## Results

###  Brain-to-Speech Reconstructs High-Quality Audio

**Figure 4:**
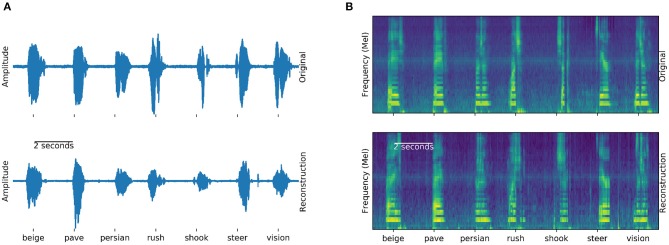
Generation example: Examples of actual (top) and generated (bottom) audio waveforms **(A)** and spectrograms **(B)** of seven words spoken by participant 5. Similarities between the generation and actual speech are striking, especially in the spectral domain **(B)**. These generated examples can be found in the Supplementary Audio 1.

The *Brain-To-Speech* approach concatenates natural units of speech and is thereby capable of creating completely unseen words, without the need to define a dictionary of recognizable words. The resulting waveforms sound very natural, as the user's own voice is employed. Many of the original spectrotemporal dynamics of speech are reconstructed. Figure 4 shows examples of generated and actual speech in audio and spectral representations. The spectral representation is only used for illustration and analysis purposes, the approach concatenated speech units in their original waveform.

We evaluated the performance of *Brain-To-Speech* for each of the six participants by computing correlations between original and generated audio spectrograms using 5-fold cross-validation. Word lists in training and test set were disjoint. Models are trained participant dependent, as brain anatomy and electrode grid locations are strongly participant dependent.

To better represent the human perception of speech, we compressed the speech spectrogram to the Mel-scale (Stevens et al., 1937) using 40 logarithmically-spaced triangular filter banks. Correlations were calculated for each mel-scaled spectral coefficient between the original and reconstruction individually and then averaged across spectral coefficients.

**Figure 5:**
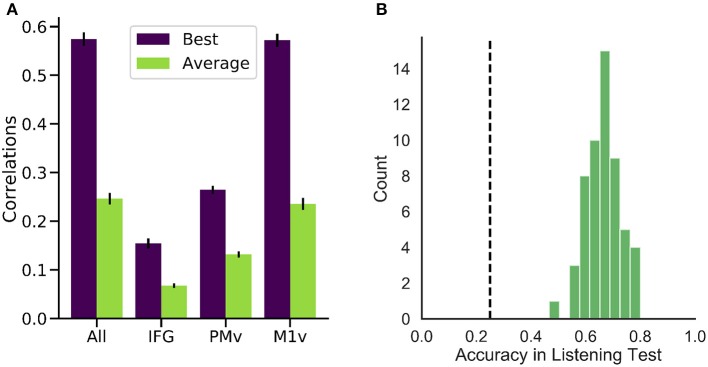
Performance of our generation approach. **(A)** Correlation coefficients between the spectrograms of original and generated audio waveforms for the best (purple) and average (green) participant. While all regions yielded better than randomized results on average, M1v provided most information for our reconstruction process. **(B)** Results of listening test with 55 human listeners. Accuracies in the 4-option forced intelligibility test were above chance level (25%, dashed line) for all listeners.

**Figure 6:**
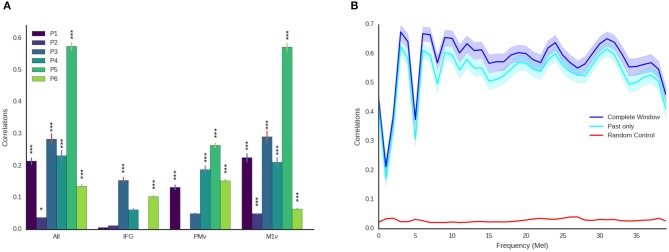
Detailed decoding results. **(A)** Correlations between original and reconstructed spectrograms (melscaled) for all participants and electrode locations. Stars indicate significance levels (^*^ Larger than 95% of random activations, ^***^ Larger than 99.9% of random activations). M1v contains most information for our decoding approach. **(B)** Detailed results for best participant using all electrodes and the entire temporal context (blue) and only using activity prior to the current moment (cyan) across all frequency coefficients. Shaded areas denote 95% confidence intervals. Reconstruction is reliable across all frequency ranges and above chance level (maximum of all randomizations, red) for all frequency ranges.

High correlations were achieved for all of the six participants (best participant *r* = 0.574 ± 0.088 STD, average *r* = 0.246 ± 0.075) when using all electrodes (Figure 5A). Intelligible speech was obtained for many examples. To establish chance level correlations, we conducted randomization tests. A randomized baseline was established by selecting random speech intervals instead of the best fitting speech unit and repeating this procedure 1,000 times for each participant. Correlation coefficients were higher than chance level for all participants when using all electrodes (highest randomized *r* = 0.04). Our reconstruction resulted in significantly higher than chance level correlations across all spectral coefficients (Figure 6B).

###  M1v Provides Most Information to Decoding Process

Examining the three functional areas of interest independently, all three regions achieved correlations above the level of random chance. Ventral primary motor cortex (M1v) clearly outperformed the other two regions (significant differences, paired *t*-test *p* < 0.001), performing almost as well as all electrodes combined (*r* = 0.235 ± 0.012). Inferior Frontal Cortex yielded lowest results of *r* = 0.067 ± 0.004. Activity from the premotor cortex yield an average of *r* = 0.132 ± 0.008.

These results show that speech motor cortex (M1v) contains most information for our reconstruction approach. Comparing the correlation coefficients for each individual participant with the randomized baseline (Figure 6A), we can see that the reconstruction using all electrodes is significantly better than chance level (*p* < 0.05) for all participants and highly significant (*p* < 0.001) for all but one participant. The reconstruction from IFG is significantly better than randomized baseline for only two participants. Information from premotor cortex (PMv) could be used to significantly reconstruct speech from 4 participants and speech motor cortex (M1v) yielded the best results with highly significant reconstruction for all 6 participants. The results for the best participant (5) show no significant difference between using all electrodes and only using information from M1v. Given the small amount of training data, the similar levels of performance between all electrodes and only M1v could also be due to the larger feature space size in the first condition.

###  Reconstructed Speech Is Intelligible

To investigate the intelligibility of the *Brain-To-Speech* approach, we conducted a listening test with 55 human listeners. The listeners were presented with individual generated audio waveforms and were required to select the most likely perceived word from a list of four word options. All listeners achieved well above chance level performance (25%) in this listening test (average of 66.1 ± 5.9%, Figure 5B).

In addition to the listening test, we calculated an objective measure of intelligibility. Our approach achieved an average Short-Term Objective intelligibility (STOI) measure (Taal et al., 2011) of 0.15, corresponding to an subjective intelligibility probability of 36%. This would mean that subjects would be able to identify the correct word in a transcription test 36% of the time. Our best participant reached a mean STOI of 0.25 corresponding to 41% intelligibility.

###  Approach Is Real-Time Ready

For future applications, it is important that our approach is real-time ready. While computing times for our limited dataset size are fast enough for real-time processing (less than 1ms for each new window every 10 ms), the long temporal context automatically induces an offset equivalent to the length of temporal context in the future. We therefore repeated our experiments using only ECoG features prior to the current time point (Figure 6B). We found that results only decreased mildly (best *r* = 0.57 for all temporal context, best *r* = 0.528 ± 0.088 using only preceding feature vectors, Figure 6B) when using no information from the future. This emphasizes that our approach can be integrated into a closed-loop system, as preceding information is sufficient to reconstruct high-quality audio.

## Discussion

*Brain-To-Speech* generated speech from the user's own voice, leading to output that sounded very natural. Reconstructed audio was of high-quality and the best examples were intelligible to human listeners. Our simple approach, based on unit selection, made no assumptions about the form, syntax or even language of the reconstructed speech. It therefore should be able to reconstruct words other than the ones used in our experiment and even sentences and continuous speech. In fact, among the words that were correctly identified by all human listeners is “Persian,” which does not follow the CVC structure. Nevertheless, *Brain-To-Speech* requires further testing with spontaneous, continuous speech in a closed-loop fashion. Our analyses are performed offline on previously collected data, but we show that *Brain-To-Speech* is capable of real-time processing, as information preceding the current moment is sufficient to generate high-quality audio. Comparing our results in terms of correlation coefficients to those achieved in the reconstruction of perceived speech from STG (Pasley et al., 2012), we achieve higher correlations for our best participants, but a lower mean *r*. However, we reconstructed articulated speech from motor areas, while Pasley et al. (2012) employed activity in auditory areas during speech perception for their approach. Martin et al. (2014) achieved higher mean correlations with their reconstruction of spectrotemporal features of speech, but lower correlations for their best participant. Their approach did not reproduce the audio waveform of the reconstruction, however. In our approach, the spectral correlations were only a secondary outcome, as we reconstructed audio waveforms directly, of which correlations were then calculated. This is distinctly different from using an approach that is directly tailored to maximize correlations.

Comparing the results of *Brain-To-Speech* to recent deep neural network based approaches (Akbari et al., 2019; Angrick et al., 2019; Anumanchipalli et al., 2019), our approach yields slightly lower correlations and STOI values, but does not require the huge computational costs of deep neural networks and is in fact fast enough for real-time processing. The formulation of our unit selection approach allows to easily integrate prior information about long term dependencies in speech and language in the future, while not requiring bi-directional processing. This can allow the *Brain-To-Speech* approach to produce good quality output with very little data, while two of the other studies (Akbari et al., 2019; Anumanchipalli et al., 2019) used significantly more data per participant.

Primary motor cortex (M1v) provided the most informative activity for decoding speech and performed as highly as electrodes from all three cortices in our best participant. Recent studies showing robot arm control in paralyzed patients (Hochberg et al., 2006) utilize electrode arrays implanted into M1 and thereby purely relying on activity generated in attempted movement. We hope that our results are also extensible to attempted speech in patients with speech deficits. It is not surprising that M1v provided the most information about speech acoustics, given recent results showing M1v contains the most information about speech kinematics (Chartier et al., 2018; Mugler et al., 2018) and results showing that speech acoustics are highly correlated with articulation (Wang et al., 2015). Additionally, our results show that high quality speech generation can be achieved with a small number of electrodes (between 12 and 20). The rapid feedback of *Brain-To-Speech* is capable of could also enable the user to learn to operate the speech prosthesis in the future, as has been demonstrated for neural upper-limb prostheses (Hochberg et al., 2006).

The intelligibility analyses indicate that the generated speech can be intelligible to human listeners despite the fact that our synthesis approach ignores semantic and linguistic knowledge. Given more training data and the opportunity for listeners to gain more experience with perceiving the idiosyncrasies of the generation, we are confident that the *Brain-To-Speech* approach would allow a BCI to generate naturalistic speech. The inclusion of prior information is known to increase the intelligibility of unit selection approaches (Hunt and Black, 1996) and could also be beneficial to our approach. In the future, a closed-loop feedback of audible speech could put the speaker in the loop, thus giving paralyzed individuals the chance to adapt their brain activity to further improve the audio output.

## Limitations

Currently, our approach relies on simultaneous recording of audible speech and ECoG activity. To adapt this approach for locked-in patients, we envision the following possibilities: Audible speech could be recorded before the patient loses the ability to speak, for example earlier in the course of a motor neuron disease. Alternatively, paralyzed patients could attempt to speak along with audio recordings of other people speaking (referred to as shadowing) and thereby generate a parallel recording of audio and brain activity data. This limitation highlights the long road toward usable BCIs based on speech processes. In the meantime, approaches based on typing activity (Pandarinath et al., 2017; Nuyujukian et al., 2018) already provide high performance communication for paralyzed patients, with an only slightly less natural paradigm.

A clear limitation of our study is the small dataset size and the intraoperative recording setup. The background noise levels and the patients' general state during an awake surgery will result in suboptimal data that are not directly transferable to the target population. However, the intraoperative setup allowed us to place the high-density grids on relevant areas for speech production and thereby investigate this process thoroughly. Longer term recordings of relevant areas, including spike recordings from intracortical arrays, are needed to bring the envisioned technology to patients. Especially recent findings of speech representations in the hand knob of the dorsal motor cortex (Stavisky et al., 2018a,b; Willett et al., 2019) might bring *Brain-To-Speech* to those in need.

Another limitation in our experimental design is the lack of control stimuli, including non-speech articulation and speech perception. The inclusion of these control stimuli in future experiments will help to identify aspects exclusive to speech production.

## Conclusion

In conclusion, we present a simple pattern matching approach for the direct synthesis of comprehensible audible speech from cortical activity in motor, premotor and inferior frontal gyri. Our approach could restore a voice and natural means of conversation to completely paralyzed patients.

## Data Availability Statement

The raw data supporting the conclusions of this manuscript will be made available by the authors, without undue reservation, to any qualified researcher.

## Ethics Statement

All participants gave written informed consent to participate in the study prior to surgery. The study design was approved by the Institutional Review Board of Northwestern University.

## Author Contributions

CH, LD, and MA analyzed the data. CH and TS evaluated the results. CH wrote the manuscript. EM, MT, and MS collected the data. EM, MT, MG, and MS designed the experiment. All authors commented on the manuscript.

### Conflict of Interest

The authors declare that the research was conducted in the absence of any commercial or financial relationships that could be construed as a potential conflict of interest.
